# Light quality determines primary production in nutrient-poor small lakes

**DOI:** 10.1038/s41598-019-41003-9

**Published:** 2019-03-15

**Authors:** Yukiko Tanabe, Makoto Hori, Akiko N. Mizuno, Takashi Osono, Masaki Uchida, Sakae Kudoh, Masumi Yamamuro

**Affiliations:** 1 National Institute of Polar Research, Research Organization of Information and Systems, 10-3 Midori-cho, Tachikawa, Tokyo, 190-8518 Japan; 2 Department of Polar Science, SOKENDAI (The Graduate University for Advanced Studies), 10-3 Midori-cho, Tachikawa, Tokyo, 190-8518 Japan; 3 Graduate School of Frontier Sciences, The University of Tokyo, 5-1-5 Kashiwanoha, Kashiwa, Chiba, 277-8563 Japan; 4 Institute for Space-Earth Environmental Research, Nagoya University, 1 Furo-cho, Chikusa-ku, Nagoya, Aichi 464-8601 Japan; 5 Department of Environmental Systems Science, Faculty of Science and Engineering, Doshisha University, 1-3 Tatara Miyakodani, Kyotanabe-shi, Kyoto, 610-0394 Japan; 6 Institute of Geology and Geoinformation, Geological Survey of Japan (GSJ), AIST, Central 7, Higashi 1-1-1, Tsukuba, Ibaraki, 305-8567 Japan

## Abstract

The availability of nutrients for primary producers has long been thought to be the main limiting factor for primary productivity in nutrient-poor lake ecosystems. However, recent studies have indicated that the availability of light energy is also important. On the other hand, the amount of phototroph was reported to decrease in summer in Antarctic lakes, furthermore, the light environment underwater was shown containing high amount of ultraviolet energy in small Antarctic lakes. Here, we hypothesized that primary productivity is limited by not only nutrients and simple light quantity but also light quality in nutrient-poor lakes. Then, we investigate factors influencing primary production by benthic phototrophic communities in shallow nutrient-poor lakes. We examine the relationships between primary production in 17 Antarctic freshwater lakes and nutrient concentrations in lake and benthic water, temperature and light energy. Primary production is decreased by ultraviolet energy reaching the lake bed, showing that production is determined by light quality. We also correlate ultraviolet energy in lake water with the catchment area of each lake. Our results show that the underwater light environment has an important influence on primary production as a key limitation factor and is sensitive to materials in runoff from the surrounding environment for pristine lakes.

## Introduction

Nutrient availability as the main limiting factor of primary productivity is an established paradigm for lake ecosystems^1–3^. However, a study of small nutrient-poor lakes showed that lake productivity, starting with benthic primary production, was not only controlled by nutrient availability but also by light attenuation caused by organic material in the lake water^4^. Another study of 11 oligotrophic lakes in the Arctic and four oligotrophic lakes in North East America showed that benthic algae, which are primarily limited by light availability, dominate primary production throughout clear-water and shallow lakes^5^.

Antarctica is one of the areas in where there are the most number of nutrient-poor lakes. Although the water in most Antarctic freshwater lakes is oligotrophic or ultra-oligotrophic, there are luxuriant benthic phototrophic mats over the lake beds^6–8^, where zooplankton predators of benthic organisms are absent or sparse^9–11^. In a study conducted in an Arctic lake, enrichment with nutrients and dissolved inorganic carbon did not enhance photosynthetic production by the benthic cyanobacterial mat, indicating that nutrients did not limit primary production by the benthos^12^. Light availability has been suggested as a controlling factor of primary productivity in Antarctic freshwater lakes, but interestingly, in a previous study the abundance of phytoplankton in the water column decreased during summer when light energy levels peaked^13^.

Previous studies have focused on simple light energy, the entire photosynthetically active radiation (PAR) spectrum or light at 440 nm, to assess factors limiting productivity. However, our previous study showed that PAR with high amount of UV radiation transmit into lake bed through water column of Antarctic oligotrophic lakes^7^. We therefore, hypothesized that not only light energy and nutrients can determine the primary productivity of primary producers but also light quality can strongly affect it in considerably nutrients-poor Antarctic lakes. To identify this hypothesis, we evaluated possibly important controlling factors such as nutrients in lake water, nutrients in interstitial water from the surface of the benthic mat, and light spectra including PAR and UV from 300–700 nm against primary productivity. The carbon stable isotope ratio δ^13^C of benthic phototrophic communities was used as an indicator of relatively representative degree of photosynthetic production^14,15^ in the calm environment of Antarctic lakes having vanishingly small dynamic force on the lakebed surface. δ^13^C of phototrophs increases with decreasing atmospheric CO_2_ concentration by kinetic isotope effect because high photosynthetic rate causes CO_2_ limitation in surrounding the cell, and consequently, relatively large amount of heavy ^13^C becomes to be taken up by phototrophs. Then, it is thought to indicate primary productivity well for longer period than photosynthetic activity which measured in momentary.

## Results and Discussion

The results for 17 Antarctic freshwater lakes are shown in Table 1. Parameters measured include the δ^13^C of organic carbon in the top 1 cm of phytobenthic mats, the dissolved inorganic nutrients phosphate and nitrogen (DIN) in whole-lake water and in interstitial water from the top 1 cm of phytobenthic mats, water temperature in summer, PAR and ultraviolet (UV) energy at the surface of the lake bed at noon on a clear midsummer day, the pH of lake water in summer and winter, and the water catchment area. The concentration of phosphate and DIN in lake water showed that all the study lakes were oligotrophic or ultra-oligotrophic. The pH of lake water in summer was considerably higher than the value measured in winter. For example, the pH of Lake Oyako was 9.0 and 6.7 in summer and winter, respectively, and the pH of Lake Misumi was 9.0 and 7.0 in summer and winter, respectively.

**Table 1:** Number, name, carbon stable isotope ratio of benthic mat surface, several environmental parameters, and key limnological variables of the study lakes.

Lake No.	Lake name	d^13^C (‰)	Lake water (mg/L)		Interstitial water (mg/L)		Temperature at maximum water depth (°C)	Light energy at maximum water depth (mW/m^2^)				pH at maximum water depth		Water catchment area (m^2^)	Maximum water depth (m)	OC (%)	TN (%)	TP (%)	C:N ratio	N:P ratio
			PO_4_^−^	DIN	PO_4_^−^	DIN		PAR	UV	PAR_S_	PAR_L_	Summer	Winter							
1	Ougi	−28.90	0.026	0.48	0.21	2.6	13	121.7	18.1	50.9	70.9	8.5	n.d.	27715	2.2	16.2	0.8	0.9	20.6	1.1
2	Hotoke	−28.22	0.076	0.59	0.11	6.3	8.7	107.0	17.3	50.1	56.9	8.6	n.d.	65026	2.8	12.2	0.6	0.7	18.8	1.1
3	Nyorai	−26.03	0.034	0.82	0.15	5.1	7.0	171.6	15.6	80.2	91.5	8.8	n.d.	67828	3.1	13.4	0.7	0.6	19.7	0.9
4	Bosatsu	−16.36	0.085	0.73	0.16	3.5	7.8	104.0	10.0	46.6	57.3	9.1	n.d.	137699	3.9	15.6	0.8	0.6	19.6	0.8
5	Jizo	−25.75	0.040	0.77	0.11	2.3	3.7	129.8	14.9	57.3	72.5	8.5	n.d.	26494	3.2	12.6	0.8	0.5	14.9	0.6
6	Kuwai	−26.85	0.050	0.54	0.12	42	2.2	113.6	12.5	50.2	63.3	8.8	n.d.	34388	3.7	7.3	0.3	1.0	22.6	3.1
7	Oyako	−14.69	0.262	0.76	1.3	160	5.9	53.4	1.4	18.9	34.6	9.1	6.7	1878902	4.0	11.6	1.3	1.5	8.9	1.2
8	Naga	−22.98	0.049	0.50	0.20	1.6	5.9	85.1	10.4	47.0	38.1	9.1	n.d.	457938	10.1	12.2	0.6	0.6	19.7	1.0
9	Oku	−24.00	0.093	0.60	0.26	28	4.3	42.9	7.0	23.2	19.8	9.0	n.d.	173591	5.7	15.7	1.3	1.0	12.1	0.8
10	Misumi	−13.15	0.059	0.46	0.18	9.1	n.d.	90.3	6.0	40.4	49.8	9.0	7.0	52731	5.5	15.1	1.1	1.5	14.1	1.4
11	Tsubaki	−12.46	0.045	0.48	0.48	6.6	3.5	10.0	0.1	3.3	6.7	9.5	6.2	4490426	4.0	7.3	0.6	2.2	12.1	3.7
12	Maruyama	−22.99	0.032	0.77	0.52	41	4.2	82.7	8.3	44.7	38.0	8.8	7.7	1427251	8.2	28.8	1.5	0.6	19.0	0.4
13	Namagi	−23.30	0.040	0.67	1.7	11	4.6	45.3	3.9	24.0	21.3	8.9	n.d.	1178656	7.4	18.3	1.4	1.1	12.9	0.8
14	Tanago	−18.30	0.055	0.71	0.31	8.6	4.2	70.6	8.1	39.0	31.6	8.8	n.d.	617681	7.9	23.5	1.5	0.6	16.0	0.4
15	Yukidori	−9.14	0.038	0.41	0.11	29	2.6	42.0	0.9	14.5	27.5	9.5	6.3	4490426	4.5	30.0	1.5	1.0	20.4	0.7
16	Hiroe	−12.99	0.050	0.90	0.19	4.6	1.6	46.4	1.9	23.2	23.2	9.8	n.d.	4490426	9.7	8.2	0.7	0.9	12.2	1.3
17	Skallen	−10.49	0.046	1.14	4.7	208	5.9	39.0	1.4	18.2	20.8	10.5	n.d.	4490426	8.7	26.5	2.5	1.2	10.6	0.5

Lake No. were shown on the study area maps (Supplymentaly Fig. -3), sampling water depths correspond to the maximum water depths, n.d. (no data), DIN (dissolved inorganic nitrogen), PAR (photosynthetically active radiation, waveband: 400–700 nm), UV (ultraviolet radiation, 300–400 nm), PAR_S_ (400–500 nm), PAR_L_ (500–700 nm), OC (organic carbon), TN (total nitrogen), TP (total phosphorus).

The δ^13^C of the aquatic moss, *Leptobryum* sp., isolated from the benthic mats in three of the study lakes, ranged from −25.8 to −30.3‰ (Table 2). The relationship between the relative electron transport rate (rETR) of photosystem II (PS II) and δ^13^C for benthic mats in ten of the study lakes is shown in Supplementary Fig. -1. The δ^13^C of the benthic mats increased with the rETR of the phytobenthic communities (*r* = 0.87, *P* = 0.001).

**Table 2:** δ13C of aquatic mosses, *Leptobryum* sp. in benthic mats.

Lake name	δ^13^C (‰)
Nyorai	−30.3 ± 0.5
Maruyama	−26.4 ± 0.3
Tanago	−25..8 ± 0.2

**Figure 1:**
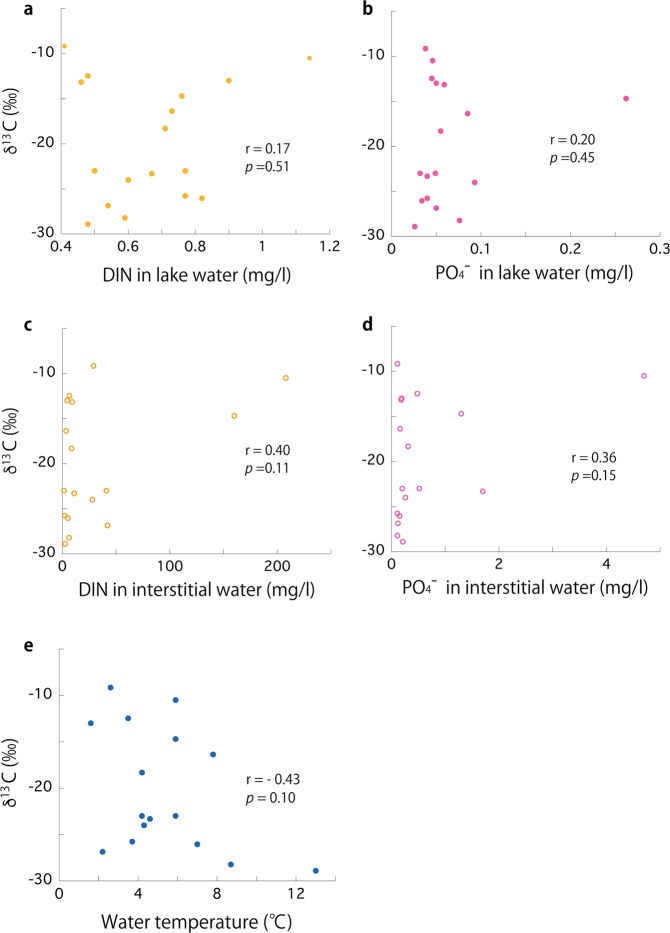
Relationship between δ^13^C of the phytobenthic mats and (**a**) dissolved inorganic nitrogen (DIN) in lake water, (**b**) phosphate in lake water, (**c**) DIN in interstitial water of the benthic surfaces, (**d**) phosphate in interstitial water of the benthic surfaces, (**e**) lake water temperature in summer for the 17 study lakes.

**Figure 2:**
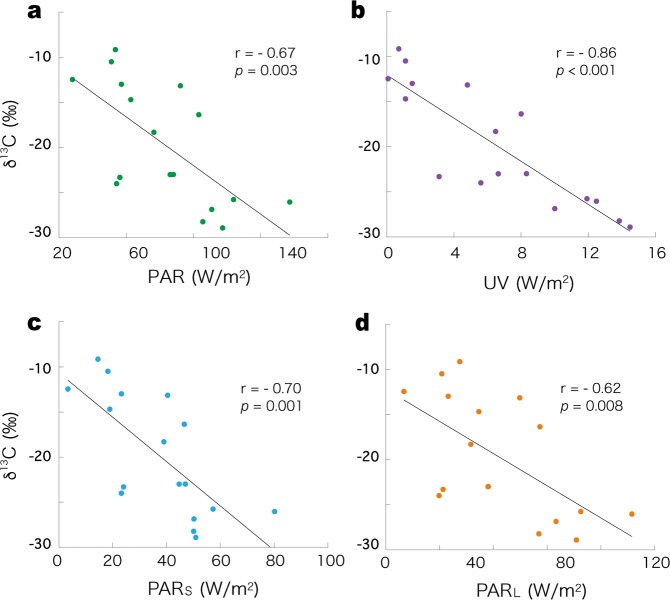
Relationship between δ^13^C of the phytobenthic mats and (**a**) photosynthetically active radiation (PAR), (**b**) ultraviolet energy (UV), (**c**) short waveband (400–500 nm) of PAR, (**d**) long waveband (500–700 nm) of PAR reaching the lake beds of the 17 study lakes.

Simple linear regression analysis showed no correlation between dissolved inorganic nutrients in lake water and δ^13^C (Fig. 1a, b), between dissolved inorganic nutrients in interstitial water from the benthic mat surface and δ^13^C (Fig. 1c, d), or between the temperature of lake water in summer and δ^13^C (Fig. 1e). However, there was a negative correlation between PAR reaching the lake beds and δ^13^C (*r* = −0.67, *P* = 0.003; Fig. 2a), and a stronger negative correlation between UV energy reaching the lake beds and δ^13^C (*r* = −0.86, *P* < 0.001; Fig. 2b). Among the wavelengths of PAR, the short waveband (PAR_S_, wavelength: 400–500 nm; *r* = −0.70, *P* = 0.001; Fig. 2c) showed stronger correlation with δ^13^C than the long waveband (PAR_L_, wavelength: 500–700 nm; *r* = −0.62, *P* = 0.008; Fig. 2d).

The results of multiple linear regression analysis for δ^13^C for each waveband of light energy reaching the lake beds are shown in Table 3. Similar to the simple linear regression analysis, an increase in UV energy was associated with a significant decrease in the δ^13^C (regression coefficient: −1.407 ± 0.331, *P* < 0.001). However, there was no correlation between PAR energy and δ^13^C (*P* = 0.064) on multiple linear regression analysis.

**Table 3:** Result in multiple linear regression analysis for δ^13^C by each waveband of light energy reaching the lakebeds.

	Regression Coefficient	SE	t-value	*p*
UV	−1.407	0.331	−4.246	<0.001
PAR	0.096	0.048	2.01	0.064066
Intercept	−15.569	1.879	−8.287	<0.0001

Multiple R-squared: 0.7545.

F-statistic: 21.52 on 2 and 14 DF, *p*-value: <0.0001.

**Figure 3:**
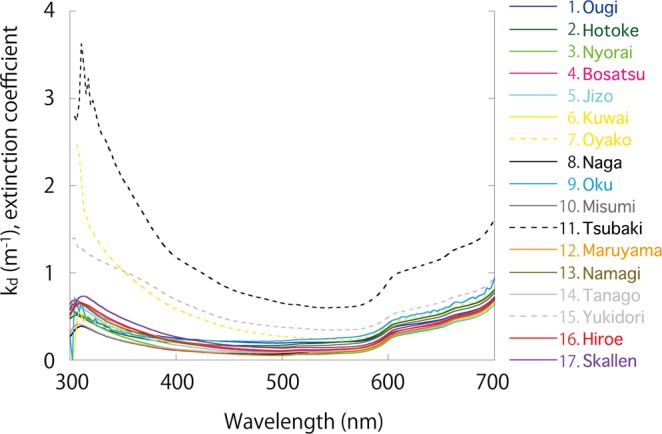
Extinction coefficient (k_d_) of light in the water column calculated using light spectra data (300–700 nm) which measured in the 17 study lakes.

The extinction coefficient k_d_ (m^−1^) of light in the water column was calculated using light spectra data (300–700 nm) for all 17 study lakes (Fig. 3). The light absorption characteristics varied among the lakes. For example, although the water in Lakes Tsubaki, Oyako and Yukidori strongly absorbed light across the 300–700 nm waveband, the wavelength distribution of absorbed light differed among the three lakes. Lake Oyako absorbed more of the short UV waveband than Lake Yukidori, but the k_d_ of Lake Oyako was less than that of Lake Tsubaki from about 350 nm to 700 nm. While the k_d_ differed among the remaining 14 study lakes, the differences were less marked compared with the three lakes mentioned above.

**Figure 4:**
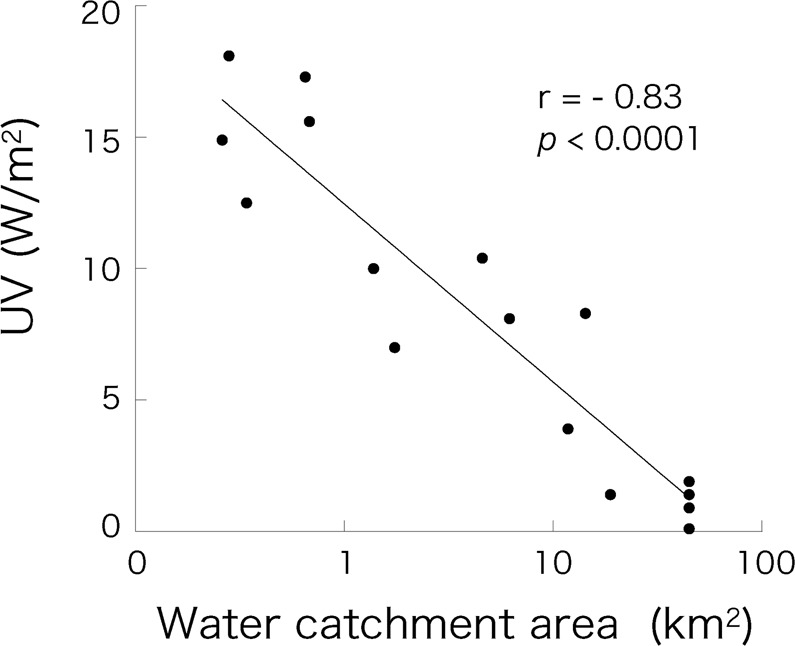
Relationship between water catchment area of the 17 study lakes and UV energy reaching the lake beds.

Across the measured light spectrum, UV energy had the strongest correlation with δ^13^C. Therefore, we investigated factors that determine the amount of UV energy that reaches lake beds. The UV energy measured at each lake bed showed a strong negative correlation to the water catchment area of that lake (*r* = −0.91, *P < *0.0001; Fig. 4).

In oligotrophic aquatic ecosystems such as those in polar regions, nutrient supply is generally thought to be the key factor limiting primary production^16,17^. Light energy has also been suggested as an essential resource for photosynthetic organisms in these oligotrophic ecosystems, where light availability may also limit primary productivity^2^. However, our study showed that larger levels of light energy reaching the lake bed, in particular UV energy, were associated with decreased primary productivity in nutrient-poor lake ecosystems. Photoinhibition is a widely accepted paradigm^18^. Active oxygen species generated by a surplus of absorbed light energy cause degradation of the reaction centre D_1_ polypeptide involved in the process of photosynthesis^19^. Photoinhibition occurs frequently in polar regions and the range of protective functions against oxidative damage may be especially important given the low ambient temperatures that would slow rates of biosynthetic repair^20,21^.

We investigated environmental factors controlling the primary production of benthic phototrophic communities in small nutrient-poor freshwater lakes using δ^13^C as an indicator of photosynthetic activity^15^. The benthic mats in the present study lakes comprised similar dominant species of cyanobacteria (*Leptolyngbya perelegans*, *Leptolyngbya tenuis*, *Nostoc* sp.), algae (*Oedogonium* sp.,) and mosses (*Leptobryum* sp.), although minor species differed among the lakes. The value of δ^13^C differed among the same species of aquatic moss (*Leptobryum* sp.) extracted from the benthic mats of three of the study lakes (Table 2). Although ETR can measure photosynthetic activity, the relationship between primary production rates and ETR is not always direct because there are the other factors and several pathways after the first step in photosystem center. However, the relationship between rETR and δ^13^C measured from phytobenthic communities collected from ten study lakes (Supplementary Fig. -1), supported the appropriateness of δ^13^C as a photosynthesis indicator, at least in the present study.

The results of the present study showed that nutrient resources did not limit primary productivity of the benthic mat communities, and showed that light energy, in particular UV energy, strongly decreased productivity. Simple linear regression analysis suggested that increased PAR decreased primary production (Fig. 2a), but this negative correlation was not maintained on multiple linear regression analysis (Table 3). However, there is a strong correlation between PAR and UV energy (Supplementary Fig. -2). Taken together, these findings suggest that the apparent decrease in primary production caused by increased PAR relates specifically to an increase in UV energy. To address the strong multicolinearity between UV energy and PAR, we further analysed the data using ridge regression and lasso regression. To estimate regression coefficients in those linear regression, a magnitude of regression coefficients as a penalty is added to error function according to regularization parameter ($\lambda$), thus the estimated coefficients necessarily prevent to multicollinearity^22,23^. We calculated a range of regularization parameter ($\lambda$) and adopted the $\lambda$ for each regression. The results supported the multiple linear regression and showed that primary production was decreased specifically by UV energy (Supplementary Table 1).

The light absorption characteristics differed among the 17 study lakes. Even when lake depth was standardized, the incident light energy reaching the lake bed differed for each lake, indicating that the PAR and UV energy reaching the lake beds was not determined by water depth alone. Lakes with a larger water catchment area showed lower levels of UV energy reaching the lake bed (Fig. 4). This finding suggests that UV energy is absorbed to a greater extent by waters that have collected from a larger catchment area. The most likely explanation is that waters from a larger catchment area contain more dissolved organic matter (DOM)^24^, and the DOM increases absorption of UV energy in the water column. The dissolved organic carbon (DOC) concentrations in Antarctic lakes are very low, ranging from 0.29 to 0.65 mg/l in the freshwater lakes of the McMurdo Dry Valley^25^ where locates in East Antarctica same as the present study area. The major carbon fractions of DOC are fluvic and hydrophilic acids and reflect a source of microbially derived components from microbial films/mats and moss communities living outside the lakes. On the other hand, the concentrations of DOC Arctic freshwater lakes are noticeably elevated compared with those of Antarctic lakes, ranging from 1.19–3.51 mg/l in polar desert, 2.33–65.50 mg/l in tundra to 4.00–16.15 mg/l in forest-tundra^7^. Moreover, the DOC concentrations in Arctic rivers are elevated compared with those of temperate rivers, and highly varied temporally^7^.

Karlsson *et al*.^4^ reported that primary productivity in their study was limited by light energy and not by nutrients, and concluded that higher levels of PAR promote high productivity in nutrient-poor lake ecosystems. The results of the present study also showed that primary productivity was limited by light and not by nutrients, but in contrast to the previous paper, higher levels of PAR decreased the productivity in nutrient-poor lakes. Bonilla *et al*.^12^ had found that post-nutrient enrichment increases in phytoplankton production that most likely driven light attenuation in water column and less light reaching the benthic communities. Carpenter *et al*.^3^ reported that coloured DOM attenuated light energy in the water column, and decreased primary production in oligotrophic lakes. These previous studies investigated energy in the PAR waveband but did not specifically investigate the UV waveband. Furthermore, these previous studies focused on Northern Hemisphere lakes. Although these lakes were oligotrophic and located in sub-Arctic regions with a cool-temperate climate, nutrients input is considerably low and UV attenuation is thought to be lower in Antarctic oligotrophic lakes because of the quite different terrestrial environment surrounding the lakes, such as there are no vascular plants, remarkable soil, and sparse animals. These are the most reason of very little plankton living in water column in Antarctic lakes, and phytoplankton bloom occurring in from temperate to most of arctic regions generally, is not caused obviously in Antarctic lakes, consequently water transparency including UV radiation is maintained at this moment. However, there is still a possibility that increase of phytoplankton causes PAR limitation to the benthic communities due to PAR and UV attenuation in water column if certain nutrients flow into the lakes by some reasons.

In conclusion, the findings of the present study support previous research that shows primary production in nutrient-poor lake ecosystems is determined by light energy rather than by nutrients. However, the novel finding of the present study is that primary production was determined by the quality of light energy rather than the quantity. The underwater environment is strongly influenced by the input of materials from the surrounding terrestrial environment, even where terrestrial vegetation is sparse as in Antarctica. Small differences in water catchment areas can have a major impact on primary production in lake ecosystems. Importantly, the productivity of lake ecosystems is strongly affected by surrounding terrestrial ecosystems and lake ecosystems are sustained in a delicate balance. Changes in material cycling can lead to changes in the light climate of lakes and can have a major impact on unproductive lake ecosystems.

## Methods

### Study area

Skarvsnes (69°20′S, 39°36′E), Langhovde (69°14′S, 39°40′E), Breidvågnipa (69°21′S, 39°48′E) and Skallen (69°40′S, 39°25′E) are ice-free areas on the Sôya Coast, eastern Lützow-Holm Bay, near Syowa station in continental Antarctica (Supplementary Fig. -3). There are more than 100 lakes in this area, varying in size, shape and limnological characteristics^7,13,26^. Most of the lakes are freshwater lakes fed by continental glaciers, although a few lakes have become saline as a result of evaporation following their isolation from the ocean during the Holocene glacio-isostatic uplift^27,28^. A total of 17 lakes in Langhovde, Skarvsnes, Skallen and Breidvågnipa were selected for the present study and shown in Supplementary Fig. -3. The number of each lake, name, sampling depth and other information are summarized in Table 1.

### Sample collection and water quality measurement

When the sampling boat drifted away from the lake centre, the exact depths of sampling points were checked using an ultrasonic depth meter (Hondex PS-7; Honda Electronics). In January–February 2010 and January–February 2012, lake benthic mats from the 17 lakes were collected by SCUBA divers or using a gravity corer. In January–February 2010, a 100-ml aliquot of lake water was collected from the mid-depth of the water column in the 17 lakes using a cylindrical water sampler (volume 500 ml). The vertical limnological profile of each lake was determined using a multiparameter water quality logger (YSI-6600V2; YSI). The water column in all lakes was vertically well mixed, and water samples were collected from the same position as the sediment cores. The water depths of the sampling points for each lake are shown in Table 1.

The top 1 cm was cut from the collected benthic mat samples from three lakes, and aquatic mosses (*Leptobryum* sp.) were separated from the samples using precise tweezers. As soon as possible after collection of the sediment core (usually within a few hours), the top 1-cm samples were separated into a solid portion and interstitial water immediately after slicing using a centrifuge (1,700 *g* for 10 min) in a field laboratory under ambient temperature conditions (3–8 °C). The separated interstitial water and the lake water samples were passed through cellulose acetate syringe filters (pore size 0.2 µm, DISMIC, Advantec). All solid and water samples were stored at −20 °C for further chemical analysis.

### Stable isotope measurement and nutrient analysis

Dissolved inorganic nutrients (PO_4_^−^, NH_4_^+^, NO_2_^−^ and NO_3_^−^) in benthic mat samples were determined colourimetrically using an AACS III autoanalyser system (Bran + Luebbe, Japan). The solid portion of the benthic mats and the aquatic moss samples were freeze-dried and ground with an agate mortar and pestle then treated with 1 N HCl in a silver cup to remove CaCO_3_^−^. Samples were combusted at 1,020 °C in an elemental analyser to determine organic C content and δ^13^C simultaneously (EA-1108 CHNS-O element analyser, Fisons Instruments). The combustion product (CO_2_) was introduced into an isotope ratio mass spectrometer (Delta Plus, Finigan) under continuous flow using He carrier gas. The ratio of ^13^C:^12^C was expressed relative to the Peedee belemnite standard for C. The ratio of ^13^C:^12^C was calculated as

1 $${\delta }^{13}C=\{R(sample)/R(standard)-1\}\times 1000\,(\textperthousand )$$

where *R* = ^13^C/^12^C. All samples were determined twice with a standard deviation (SD) of ±0.11 for *n* = 11 for δ^13^C.

### Measurement and calibration of light spectra and calculation of water catchment area

A spectral irradiance sensor equipped with a depth sensor (RAMSES-UV/VIS, TriOS) was used to measure light from 300 to 700 nm. A series of triplicate measurements were made above the lake surface, just below the surface and at the lake bed within 5 min at the same locations where the benthic mat samples were collected, and the data for each measurement location were averaged. To account for changes in the light spectra related to weather and the time of measurement, the attenuation coefficient, *k*_*d*_ (m^−1^) was calculated as

2 $${k}_{d}=-\,{d}^{-1}\times ln\{{I}_{d}(\lambda )/{I}_{{0}}({\lambda })\}$$

where *d* (m) is the water depth of each lake bed, *I*_*d*_ (W) is the light intensity observed at the water depth of each lake bed, and *I*_*0*_ (W) is the light intensity observed just below the surface of the lake. Maximum radiation reaching each lake bed *I*_*d*_′ (W) was calibrated by using the *k*_*d*_ and a standard value of ground radiation, the value at noon on a midsummer day under clear sky, as follows:

3 $${I}_{d}^{\prime} (\lambda )={I}_{s}(\lambda )\times exp({k}_{d}\times d)$$

where *I*_*s*_ (W) was light intensity reaching the ground at noon on a midsummer day under clear sky conditions in the study area. Maximum radiation *I*_*d*_′*(λ)* was separated into UV radiation (waveband 300–400 nm), PAR (waveband 400–700 nm), PAR_S_ (waveband 400–500 nm) and PAR_L_ (waveband 500–700 nm). The water catchment area of each lake was calculated from pixel size on a topographic map of the region using Adobe Photoshop CS6.

### Measurement of photosynthetic activity

Photosynthetic yield (PS II quantum yield) of the benthic mats from the ten lakes was measured using a PAM chlorophyll fluorometer (Water-PAM, Walz) with control and analysis software, Win-control. To determine the light-photosynthetic rate (the rETR, μmol photon m^−2^ s^−1^) measurements were made under nine stepwise actinic light intensities (0, 34, 50, 77, 112, 171, 253, 353 and 470 mmol photons m^−2^ s^−1^ of PAR with a 30 s duration) and >2,000 mmol photons m^−2^ s^−1^ of saturating pulse with a 0.4 s duration. The gain value of the photoelectric multiplier (PM-Gain) was set to 3 throughout the measurements. A small piece of samples was placed into a measuring cuvette of the fluorometer, which was filled with a few ml of filtered lake water collected from the same lake. Light curves were obtained by running a rapid light curve protocol in the Win-control software. The photosynthetic rate was expressed as the *rETR*^29^ as follows:

4 $$rETR=(Fm^{\prime} -F)/Fm^{\prime} \times PAR$$

where *F* and *Fm*′, respectively, are the transient and maximum fluorescence levels at a certain actinic light intensity at a given time. *Fm*′ *−* *F)/Fm*′ indicates the PS II yield and PAR (mmol photons m^−2^ s^−1^) is photosynthetically active radiation.

The data were exported into Kaleida-Graph software (ver. 3.5 for Windows), and were fitted to the following equation^18,30^ using a Levenberg–Marqurdt regression algorithm as follows:

5 $$rETR=PAR/(aPA{R}^{{2}}+bPAR+c)$$

where *a*, *b* and *c* are regression coefficients to fit to the curve. The photosynthetic parameters were calculated as

6 $$rET{R}_{max}={[b+{2}{(ac)}^{{0.5}}]}^{-{1}}$$

where *rETR*_*max*_ (μmol photon m^−2^ s^−1^) represents the maximum rETR as described by Ban *et al*.^31^.

## Supplementary information

Supplementary information
