# Supplementary material for: Light quality determines primary production in nutrient-poor small lakes
Source: Sci Rep. 2019 Mar 15;9:4639. doi: 10.1038/s41598-019-41003-9 (PMC6420600; doi:10.1038/s41598-019-41003-9)

Light quality determines primary production in nutrient-poor small lakes

Yukiko Tanabe^1,2*^, Makoto Hori^3^, Akiko N. Mizuno^4^, Takashi Osono^5^, Masaki Uchida^1,2^, Sakae Kudoh^1,2^ & Masumi Yamamuro^3,6^

^1^National Institute of Polar Research, Research Organization of Information and Systems, 10-3 Midori-cho, Tachikawa, Tokyo 190-8518, Japan. ^2^Department of Polar Science, SOKENDAI (The Graduate University for Advanced Studies), 10-3 Midori-cho, Tachikawa, Tokyo 190-8518, Japan. ^3^Graduate School of Frontier Sciences, The University of Tokyo, 5-1-5 Kashiwanoha, Kashiwa, Chiba 277-8563, Japan. ^4^Institute for Space-Earth Environmental Research, Nagoya University, 1 Furo-cho, Chikusa-ku, Nagoya, Aichi 464-8601, Japan. ^5^Department of Environmental Systems Science, Faculty of Science and Engineering, Doshisha University, 1-3 Tatara Miyakodani, Kyotanabe-shi, Kyoto 610-0394, Japan. ^6^ Institute of Geology and Geoinformation, Geological Survey of Japan (GSJ), AIST, Central 7, Higashi 1-1-1, Tsukuba, Ibaraki, 305-8567, Japan.

*Correspondence should be addressed to Y.T (email: ukko@nipr.ac.jp).

Tel: +81-42-512-0736; Fax: +81-42-528-3492.

**Supplementary Information**

**Supplementary Table**

Table S1. Result in ridge and lasso regression analysis for δ^13^C by each waveband of light energy reaching the.

**Supplementary Figures**

Supplementary Figure-1. Relationship between δ^13^C and relative electron transport rate of photosystem II for benthic mats surface in ten of the study lakes that were measured in the field camp.


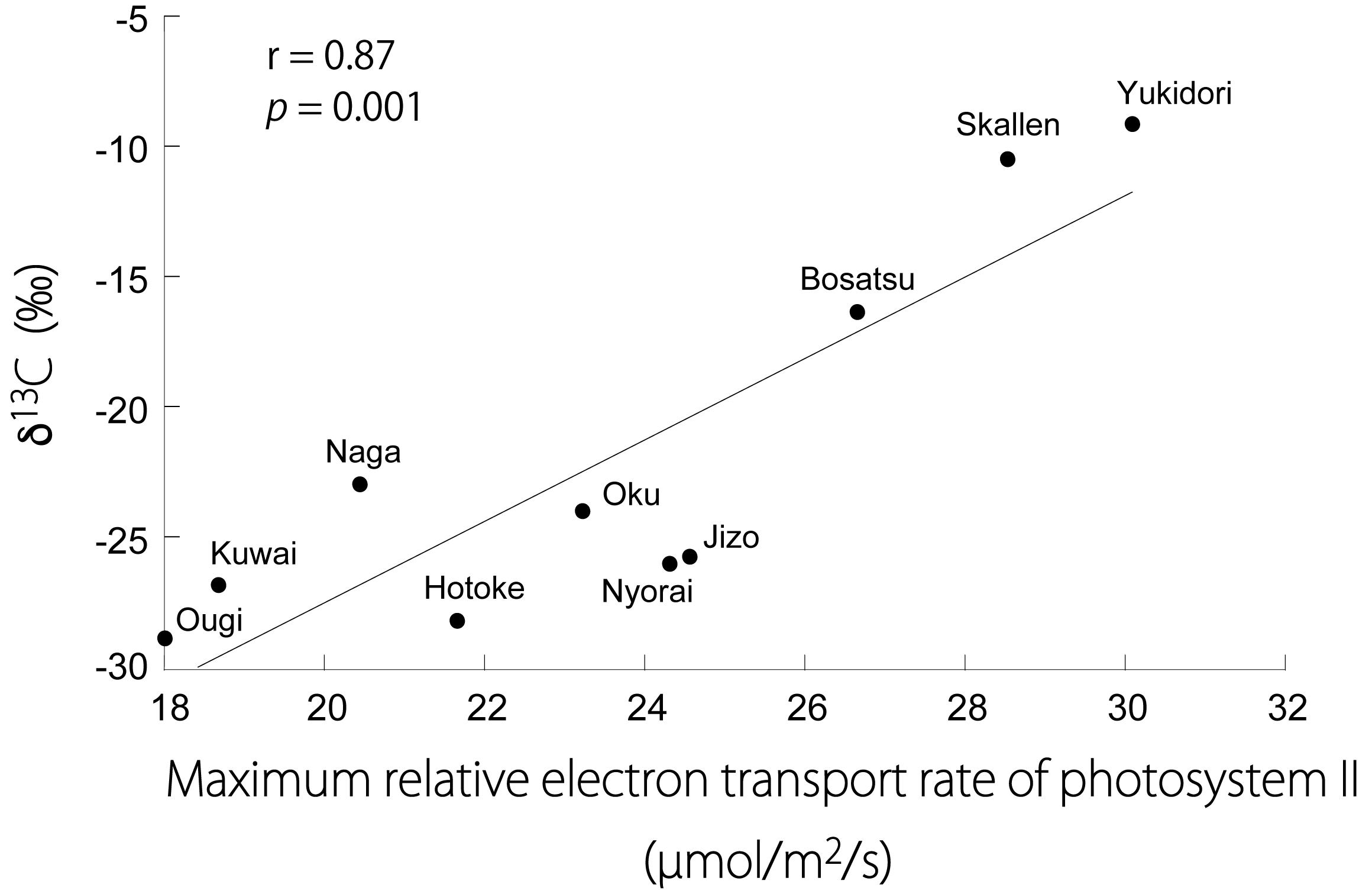


Supplementary Figure-2. Relationship between UV energy and PAR reaching the lake beds of the 17 study lakes.


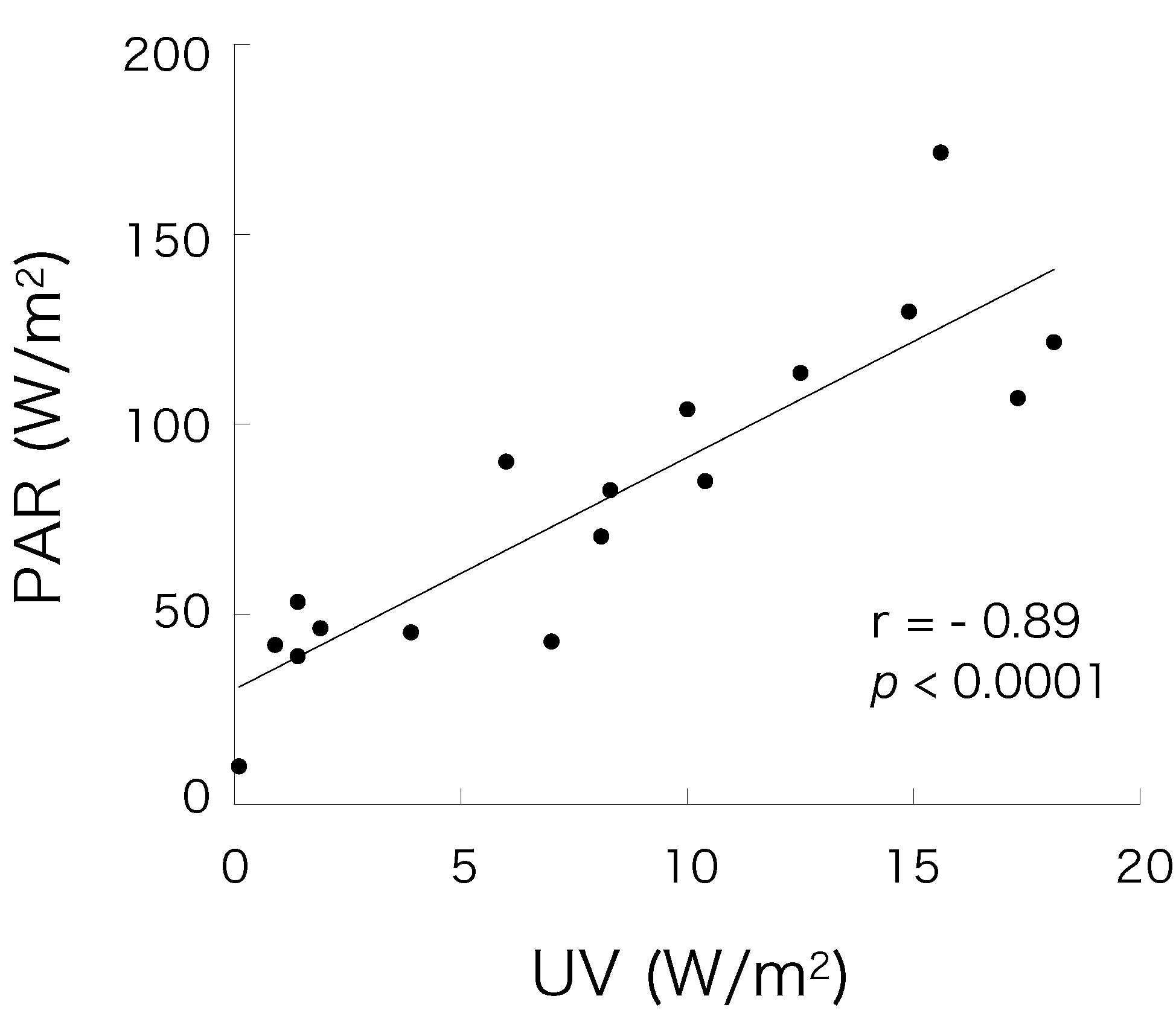


Supplementary Figure-3. Maps of study areas and the 17 lakes. The numbers on the maps correspond to “Lake No.” of Table 1.


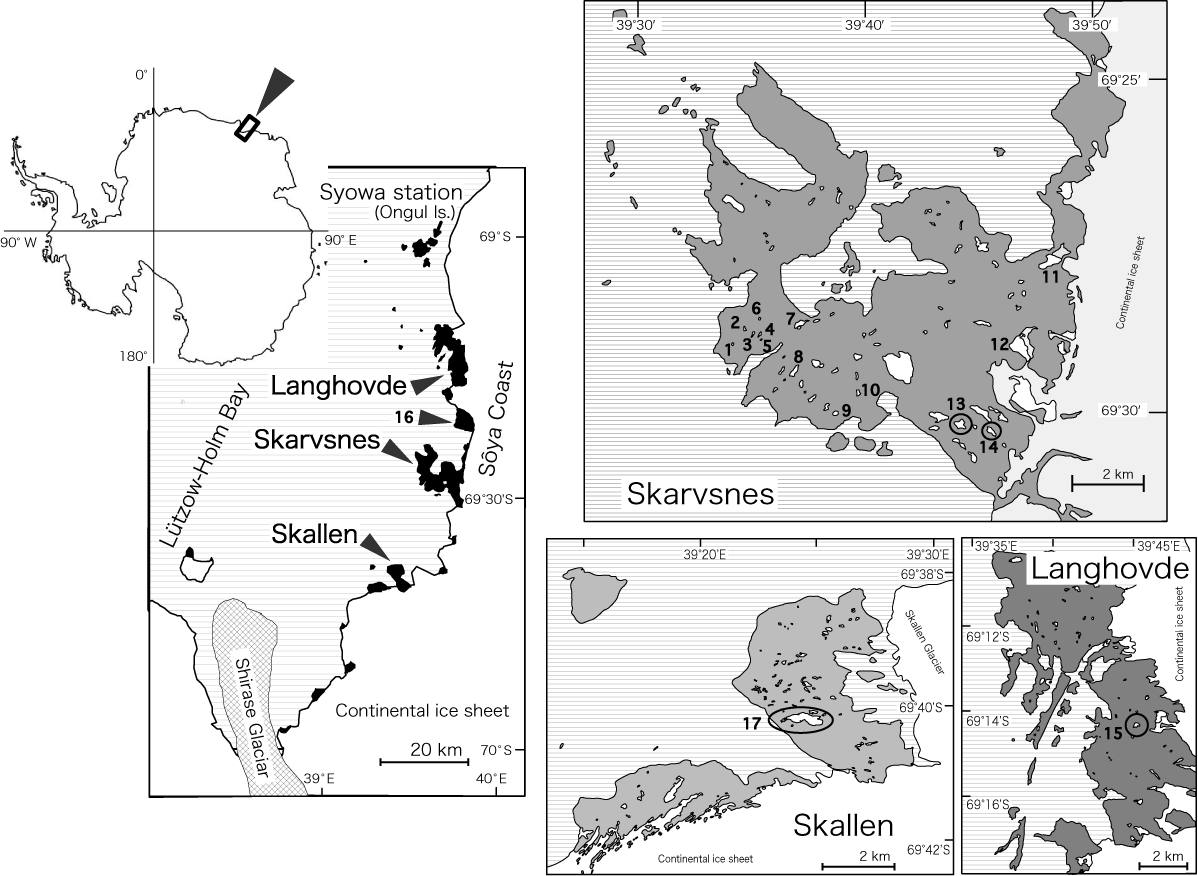

Supplement: Supplementary file 1 — Supplementary information [file 41598_2019_41003_MOESM1_ESM.doc]
